# Fusion of fNIRS and fMRI data: identifying when and where hemodynamic signals are changing in human brains

**DOI:** 10.3389/fnhum.2013.00676

**Published:** 2013-10-16

**Authors:** Zhen Yuan, JongChul Ye

**Affiliations:** ^1^Bioimaging Core, Faculty of Health Sciences, University of MacauMacau SAR, China; ^2^Department of Bio and Brain Engineering, KAISTDaejeon, South Korea

**Keywords:** fMRI, fNIRS, independent component analysis, multimodal imaging methods, cognitive neurosciences

## Abstract

In this study we implemented a new imaging method to fuse functional near infrared spectroscopy (fNIRS) measurements and functional magnetic resonance imaging (fMRI) data to reveal the spatiotemporal dynamics of the hemodynamic responses with high spatiotemporal resolution across the brain. We evaluated this method using multimodal data acquired from human right finger tapping tasks. And we found the proposed method is able to clearly identify from the linked components of fMRI and fNIRS where and when the hemodynamic signals are changing. In particular, the estimated associations between fNIRS and fMRI will be displayed as time varying spatial fMRI maps along with the fNIRS time courses. In addition, the joint components between fMRI and fNIRS are combined together to generate full spatiotemporal “snapshots” and movies, which provides an excellent way to examine the dynamic interplay between hemodynamic fNIRS and fMRI measurements.

## Introduction

Functional near infrared spectroscopy (fNIRS) offers unsurpassed high temporal resolution and provides quantitative hemodynamic information for both oxyhemoglobin (*HbO*_*2*_) and deoxyhemoglobin (*HbR*), which plays an important role in the *in vivo* study of cognitive processing in the human brain (Jobsis, 1977; Cope and Delpy, 1988; Hoshi, 2003; Singh et al., 2005; Huppert et al., 2009; Ye et al., 2009; Yuan et al., 2010a,b; Brunno et al., 2011; Egetemeir et al., 2011; Gagnon et al., 2012; Yuan, in press). Similar to its fNIRS counterpart, functional magnetic resonance imaging (fMRI) is also a non-invasive imaging method that measures the hemodynamic responses to even-related neural activity with excellent spatial resolution and low temporal resolution. To take advantages of the complementary information from these two imaging modalities, two broad methods for fNIRS and fMRI/MRI integration have been developed for different clinical cases: (1) “spatial constraint,” in which spatial information from fMRI/MRI images are utilized to aid diffuse optical imaging based on fNIRS measurements (Carpenter et al., 2007; Ferradal et al., 2013); (2) “temporal correlation,” where fMRI bold signals are processed to generate the correlation with *HbO_*2*_* and *HbR* concentration changes converted from fNIRS recordings (Cui et al., 2011; Tak et al., 2011; Gagnon et al., 2012). However, so far the linking between *HbO_*2*_*/*HbR* signals and fMRI spatial maps has not been extensively investigated, which represents one of the main challenges for fNIRS and fMRI fusion. Therefore, it is crucial for us to develop new imaging techniques to reveal the connections between these two measurements so that we are able to examine the dynamic interplay between space and time of hemodynamic responses with high spatiotemporal resolution.

Interestingly joint independent component analysis (jICA) method has been developed to compute the linked temporally independent event-related potential (ERP) components and spatial independent fMRI components, which enables inferences to be made using estimated associations between fMRI sources and ERP electromagnetic sources (Calhoun et al., 2006a,b; Sui et al., 2009). However, what has not been tried is a joint estimation of the temporal parts of fNIRS waveforms and the spatial maps of fMRI images. In this study, the jICA is extended to identify the spatiotemporal decompositions composed of fMRI spatial components indicating where the hemodynamic signals are changing and fNIRS components indicating when the hemodynamic signals are changing. The fNIRS-fMRI fusion method will involve calculating for given stimuli, the connection between the time-locked fNIRS waveforms and fMRI activation maps for all participants or different measurement sections from a single subject. It is anticipated that the results derived from jICA could be visualized by computing spatiotemporal “snapshot,” which provides an effective way to examine the dynamic interplay between fNIRS and fMRI hemodynamic sources.

## Methods

In terms of jICA, the joint spatial and temporal independences of fNRIS and fMRI are assumed to satisfy the following generative model for the data (Calhoun et al., 2006a,b),

(1) $$X^{\text{fNIRS}}=AS^{\text{fNIRS}},X^{\text{fMRI}}=AS^{\text{fMRI}}$$

in which ***X***^fNIRS^ is the group data from the chromophore concentration change of *HbO_*2*_* or *HbR* for *n* subjects/*n* sections of a single subject and ***X***^fNIRS^ = [*X*^fNIRS^_1_, *X*^fNIRS^_2_, … *X*^fNIRS^_*n*_]^*T*^, ***X***^fMRI^ is the group data from fMRI for *n* subjects/*n* sections of a single subject and ***X***^fMRI^ = [*X*^fMRI^_1_, *X*^fMRI^_2_, … *X*^fMRI^_*n*_]^*T*^, ***S***^fMRI^ are the fMRI sources and ***S***^fMRI^ = [*S*^fMRI^_1_, *S*^fMRI^_2_, … *S*^fMRI^_*n*_]^*T*^, and *S*^fNIRS^ are the fNIRS sources and *S*^fNIRS^ = [*S*^fNIRS^_1_, *S*^fNIRS^_2_, … *S*^fNIRS^_*n*_]^*T*^. The shared lined mixing matrix ***A*** are written,

(2) $$A=\left[\begin{array}{cccc} a_{11} & a_{12} & \ldots & a_{1n}\\ a_{21} & a_{22} & \ldots & a_{2n}\\ . & . & . & .\\ a_{n1} & a_{n2} & \ldots & a_{nn} \end{array}\right]$$

It is note Equation 1 can be rewritten as a single matrix equation,

(3) $$\left[\begin{array}{ll} X_{1}^{\text{fNIRS}} & X_{1}^{\text{fMRI}}\\ X_{2}^{\text{fNIRS}} & X_{2}^{\text{fMRI}}\\ \ldots & \ldots \\ X_{n}^{\text{fNIRS}} & X_{n}^{\text{fMRI}} \end{array}\right]=A\left[\begin{array}{cc} S_{1}^{\text{fNIRS}} & S_{1}^{\text{fMRI}}\\ S_{2}^{\text{fNIRS}} & S_{2}^{\text{fMRI}}\\ \ldots & \ldots \\ S_{n}^{\text{fNIRS}} & S_{n}^{\text{fMRI}} \end{array}\right]$$

We employ the infomax ICA method for jICA of Equation 3, which utilizes a gradient ascent iteration algorithm to maximize the entropy of the output of a single layer neural network (Bell and Sejnowski, 1995). The resulting updated equation for the algorithm to calculate the shared unmixing matrix ***W*** (i.e., the inversion of ***A***), the fused independent fNIRS sources ***u***^fNIRS^ and fMRI sources ***u***^fMRI^ is as follows,

(4) $$\Delta w=\eta \left\{I-2y^{\text{fNIRS}}{\left(u^{\text{fNIRS}}\right)}^{T}-2y^{\text{fMRI}}{\left(u^{\text{fMRI}}\right)}^{T}\right\}W$$

in which ***y***^fNIRS^ = *g*(***u***^fNIRS^), ***y***^fMRI^ = *g*(***u***^fMRI^), ***u***^fNIRS^ = ***WX***^fNIRS^, ***u***^fMRI^ = ***WX***^fMRI^, and *g*(*x*) = 1/(1+e^−*x*^) is the non-linearity in the neural network. The initial value for ***W***, ***W***(0) is a matrix composed of random vectors.

The jICA method generally doesn't provide us the details on how the components between fNIRS and fMRI interact with one another. To achieve this, the spatiotemporal “snapshots” of the most significant components are generated in two ways (Calhoun et al., 2006a,b). First we need to calculate the linear combination of the fMRI components weighted by their joint fNIRS time courses for a specific point in time. If the *n* spatial (fMRI) and temporal (fNIRS) components are given by ***S*** = [***s***_1_ ***s***_2_ … ***s***_*n*_], and ***T*** = [***t***_1_ ***t***_2_ … ***t***_*n*_], where ***t***_*i*_ is a *T* × 1 vector containing the fNIRS time points and ***s***_*i*_ is a *V* × 1 vector represents the *V* brain voxels, the fMRI movie is computed as ***M***_fMRI_ = |***T***| × ***S***^*T*^. It is noted the absolute value is utilized here because the joint components are fused using a single parameter. And a change in the amplitude of the fMRI component is directly linked to the change in the fNIRS component by this parameter. Meanwhile the fNIRS movie is estimated by ***M***_fNIRS_ = ***T*** × |***S***|^*T*^, in which the time course for a given fMRI voxel is computed.

## Results

### Behavior tasks and fNIRS-fMRI recordings

**Figure 1:**
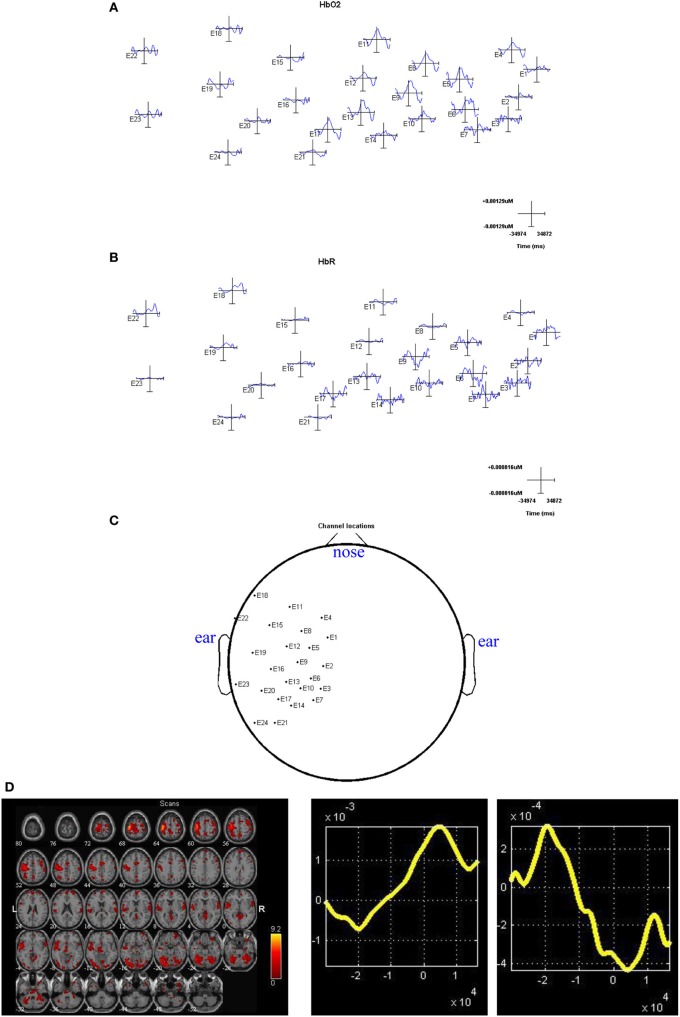
**(A)** The data review for *ΔHbO_*2*_* measurements; **(B)** the data review for *ΔHbR* measurements; **(C)** Channel configurations along the scalp for right finger tapping tasks; **(D)** the section averaged fMRI image (left side), *ΔHbO_*2*_* results (middle) and *ΔHbR* results(right side). The axes (bottom middle and bottom right) illustrate the time scale, in ms, whereas the scale for the middle and right columns records chromophore concentrations in μM.

The fNIRS tests are implemented with a block design for a right finger tapping task. The experiment is performed with a 24-chnnel fNIRS system (Oxymon MKIII, Artinis), which has 8 sources, 8 detectors, and 24 channels. In this system, two continuous wave lights at wavelengths 781 and 856 nm are emitted at each source fiber. In the case of block design for right finger tapping tasks, the onset time for the first trigger was at 42 s, then followed by a 21 s period of activation alternated with a 30 s period of rest. This was repeated 10 times for the subject. As such, the total recording time was 552 s. During the task period, subject was instructed to perform a finger flexion and extension action repeatedly. Data segmentation, which is also known as epoching in signal processing, is utilized to chop up the continuous fNIRS data into small time periods. The general way to do this is to extract segments surrounding the event codes from the experiments, e.g., from −35 s prior to the event onset until 35 s after the event code in this study. The original photon density datasets could be downloaded from (http://bisp.kaist.ac.kr/NIRS-SPM/Sample_data). The converted *ΔHbO_*2*_* and *ΔHbR* measurements (the unitless differential path length factor *DPF* = 4; sampling rate: 9.75 Hz) are filtered, segmented and plotted in Figures 1A,B, respectively. The configurations of 24 channels located on the scalp are also provided in Figure 1C. We found channels 8–12 are very unique because their locations are close to the left motor cortex so that they are able to reveal the hemodynamic responses from both the right finger tapping stimuli and other events. As a result, signal averaging is implemented for the datasets from these five channels to generate the representative *ΔHbO_*2*_*/*ΔHbR* measurements with reduced noise. In particular, five-section (runs 2, 4, 6, 8, and 10) measurements are selected from the representative ten-run fNIRS recordings for further fusion analysis since they show the best signal noise ratio during the stimulus processing.

For fMRI recordings, the fiber length was 10 m to connect the optodes in the MR scanner to the NIRS instrument in the MR control room. A 3.0 T MRI system (ISOL, Republic of Korea) was used to measure the BOLD response. During the blocked task paradigm, the echo planar imaging (EPI) sequence was used with *TR*/*TE* = 3000/35 ms, flipangle = 80°, 35 slices, and 4 mm slice thickness (Ye et al., 2009). The fMRI data could be downloaded partly from the same website for the same subject or requested from the KAIST lab. Based on the datasets provided, we generated five mean MRI images which are considered as five-section fMRI measurements (Calhoun et al., 2001; Ye et al., 2009).

Then we will combine the five-section averaged fNIRS recordings in temporal domain and five mean fMRI images in space to extract the joint independent components for hemodynamic fusion. It should be noted here the mean fMRI images are not generated from the raw fMRI images at each TR. As such, the fMRI image for each section may not accurately match its correlated fNIRS measurements in terms of recording times, which may have some influence on the final results. However, the influences should not be that significant since we use mean data from each section for fusion. The five-section averaged fMRI image and *ΔHbO_*2*_*/*ΔHbR* results are presented in Figure 1D for references.

### Results of fNIRS-fMRI fusion

**Figure 2:**
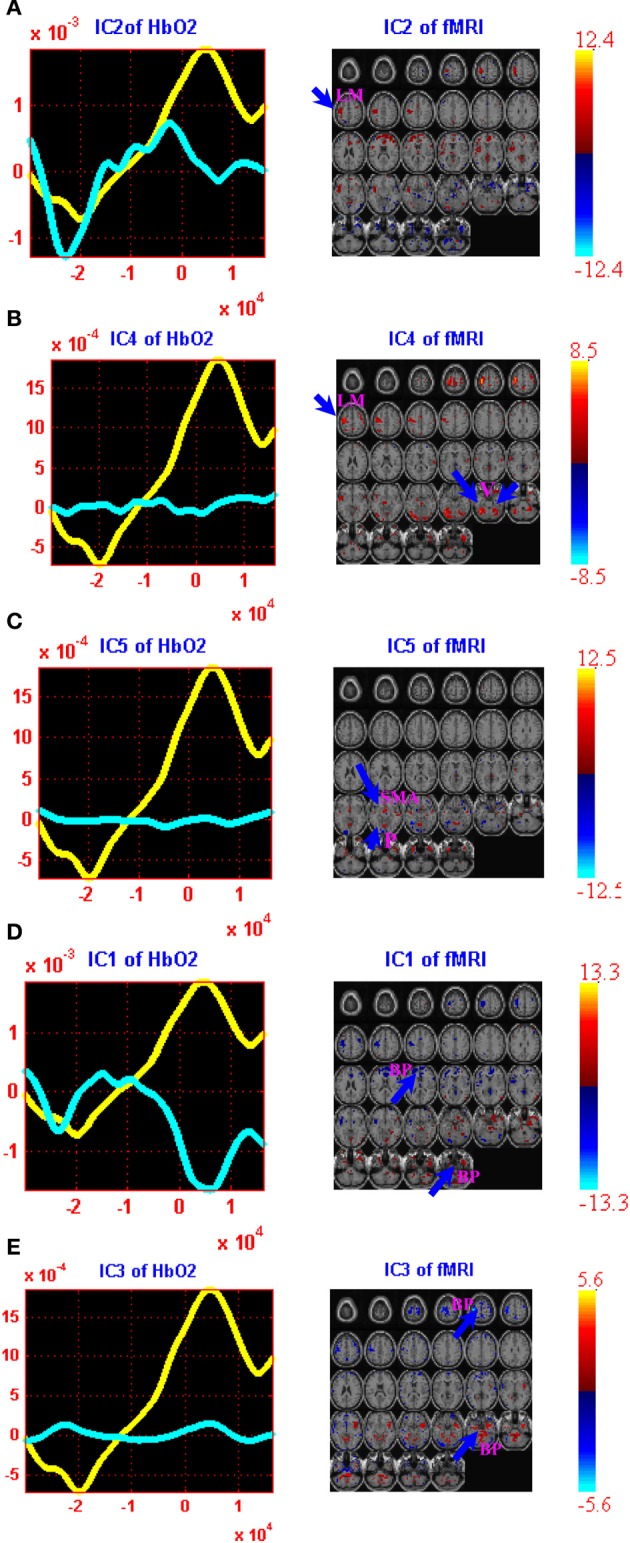
**jICA decomposition of *ΔHbO_*2*_* and fMRI joint data for right finger tapping tasks: three components were found to be significantly correlated with finger tapping tasks [Independent components (ICs) 2, 4, and 5] while two were well-correlated with the physiology and body movement noise (ICs 1 and 3).** Each of these components is shown in a separate panel in the figure: IC2 **(A)**, IC4 **(B)**, IC5 **(C)**, IC1 **(D)** and IC3 **(E)**. The fMRI maps are thresholded at |*Z*| > 1.5 for display purposes. The averaged event-related *ΔHbO_*2*_* time course is shown in yellow (the same for all figures) and the *ΔHbO_*2*_* component is plotted in cyan. Positive (orange) and negative (blue) *Z*-values are shown in the image. The axes (bottom) illustrate the time scale, in ms, whereas the scale (left) records chromophore concentrations in μM for the figures on the left column. The scale for the figures on the right column shows the bold signal intensity. LM, Left primary motor cortex; SMA, Supplementary motor area; P, Parietal cortex; V, Visual cortex; BP, Body movement/physiology noise.

**Figure 3:**
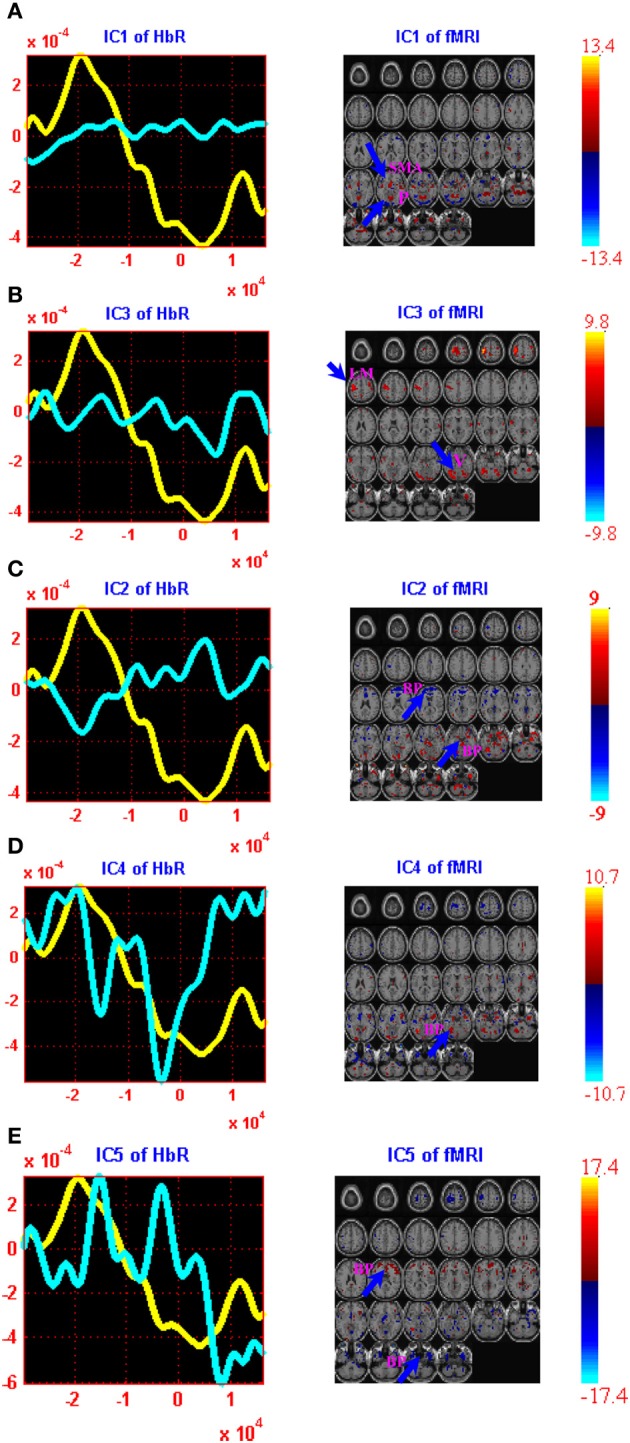
**jICA decomposition of *ΔHbR* and fMRI joint data for right finger tapping tasks: two components were found to be significantly correlated with right finger tapping task (ICs 1 and 3) while three were well-correlated with the physiology and body movement noise (ICs 2, 4, and 5).** Each of these components is shown in a separate panel in the figure: IC1 **(A)**, IC3 **(B)**, IC2 **(C)**, IC4 **(D)** and IC5 **(E)**. The fMRI maps are thresholded at |*Z*| > 1.5 for display purposes. The averaged event-related *HbR* time course is shown in yellow (same for all figures) and the *ΔHbR* component is plotted in cyan. Positive (orange) and negative (blue) *Z*-values are shown in the image. The axes (bottom) illustrate the time scale, in ms, whereas the scale (left) records chromophore concentrations in μM for the figures on the left column. The scale for the figures on the right column shows the bold signal intensity. LM, Left primary motor cortex; SMA, Supplementary motor area; P, Parietal cortex; V, Visual cortex; BP, Body movement/physiology noise.

The computed joint components are provided in Figures 2, 3, in which the spatial components and regions of brain activity from the fMRI maps are plotted on the right while the correlated temporal joint components along with the mean *ΔHbO_*2*_*/*ΔHbR* signals are given on the left. We can see from Figures 2, 3 that the time courses of temporal *ΔHbO_*2*_*/*ΔHbR* components correspond well to different peaks on the mean *ΔHbO_*2*_*/*ΔHbR* signals. Interestingly, when put together the fMRI maps and fNIRS signals, we found the five spatial joint components correlate very well with the five temporal joint components. For example, the first negative peak of *ΔHbO_*2*_* in Figure 2A basically identifies the physiology noise such as visual activity and eye blinks. Also visible for this waveform of the second temporal joint component in Figure 2A is a late positive peak. During the positive peak, the hemodynamic activity is mainly found in the left motor cortex. Then the joint components from *ΔHbO_*2*_*/*ΔHbR* and fMRI are combined together to generate full spatiotemporal movies to more clearly display the dynamic interplay between the fNIRS and fMRI measurements. In particular, two movies are created to show the spatiotemporal dynamics of the fMRI and *ΔHbO_*2*_* fusion, in which Movie 1 displays the hemodynamic responses of right finger tapping events (composite spatiotemporal joint components 2, 4, and 5) while Movie 2 basically reveals brain activity from physiology and body movement(composite joint components 1 and 3). The spatiotemporal hemodynamic changes derived from fMRI and *ΔHbR* fusion are also recorded by Movies 3, 4 for right finger tapping tasks (composite joint component 1 and 3) and physiology and body movement noise (composite joint component 2, 4, and 5), respectively.

**Figure 4:**
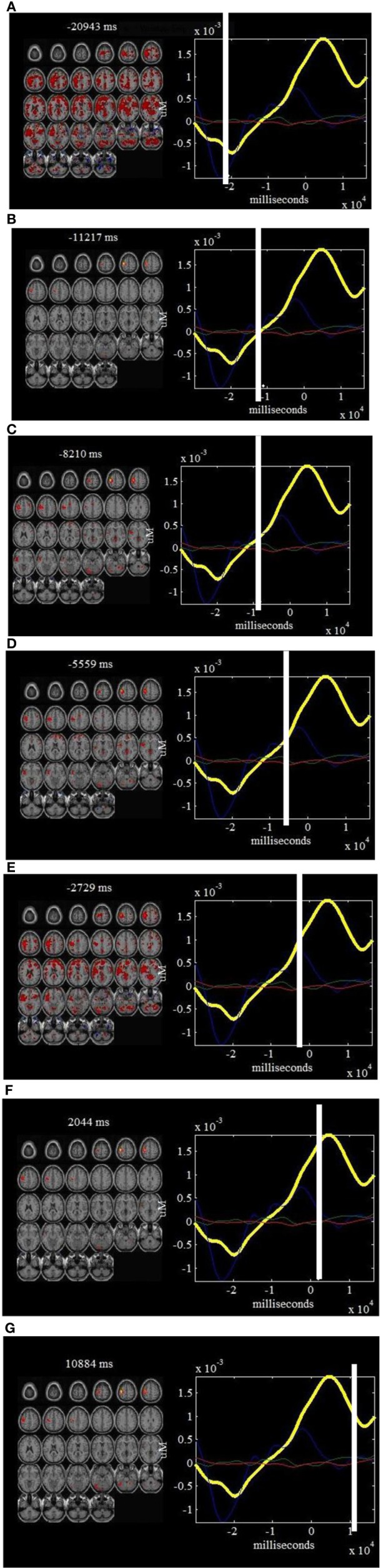
***ΔHbO*_*2*_ components and fMRI “snapshots”: on the left of each window is shown a linear combination of the fMRI maps that are well-correlated with finger tapping tasks (composite ICs 2, 4, and 5), weighted by the *ΔHbO_*2*_* part of the components at a specific point in time.** On the right of each window is shown the estimated *ΔHbO_*2*_* components that are correlated with right finger tapping tasks. The time courses for IC2 (in blue), IC4 (in green) and IC5 (in red) are also plotted on the right of each window. Such a display provides a dynamic way to visualize the brain activity at different time points: −20943 ms **(A)**, −11217 ms **(B)**, −8210 ms **(C)**, −5559 ms **(D)**, −2729 ms **(E)**, 2044 ms **(F)** and 10884 ms **(G)**.

**Figure 5:**
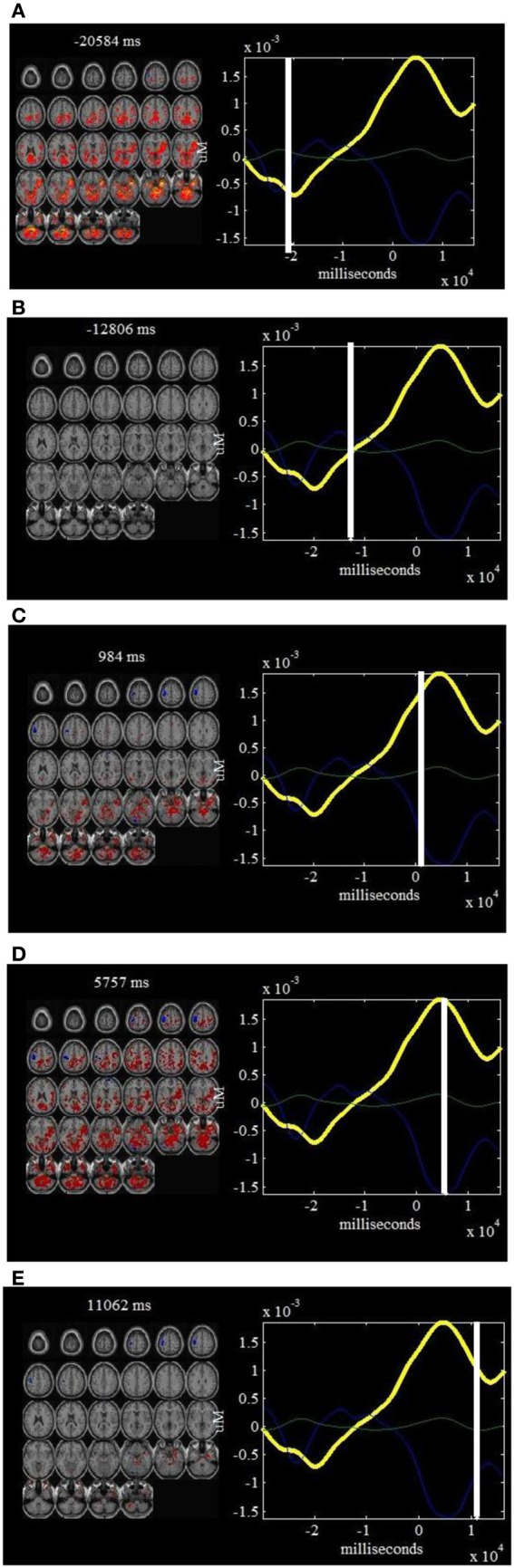
***ΔHbO_*2*_* components and fMRI “snapshots”: on the left of each window is shown a linear combination of the fMRI maps that are well-correlated with physiology and body movement noise (composite ICs 1 and 3), weighted by the *ΔHbO_*2*_* part of the components at a specific point in time.** On the right of each window is shown the estimated *ΔHbO_*2*_* components that are correlated physiology and body movement noise. The time courses for IC1 (in blue) and IC3 (in green) are also plotted on the right of each window. Such a display provides a dynamic way to visualize how the noise affects the brain activity at different time points: −20584 ms **(A)**, −12806 ms **(B)**, 984 ms **(C)**, 5757 ms **(D)** and 11062 ms **(E)**.

**Figure 6:**
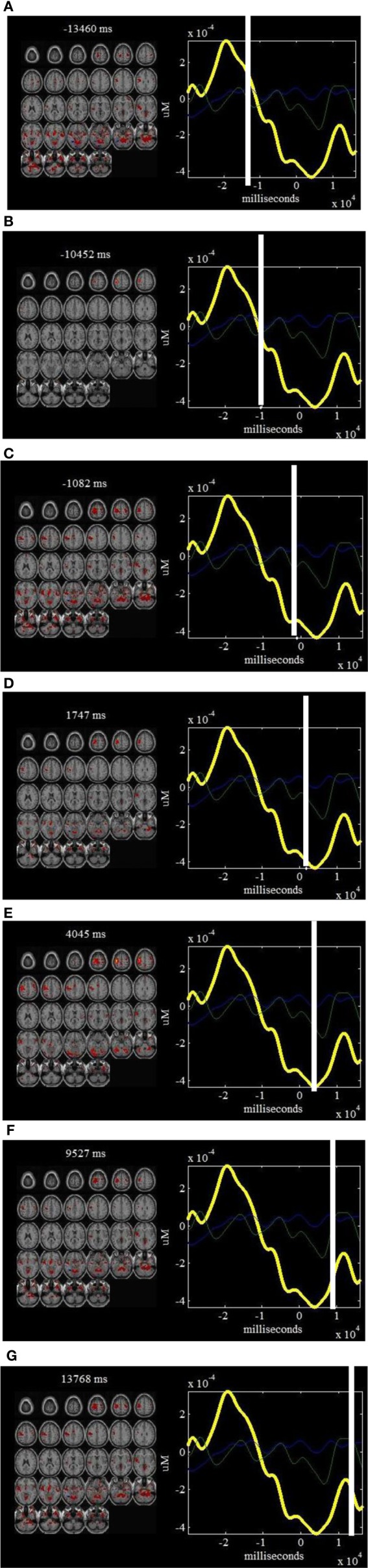
***ΔHbR* components and fMRI “snapshots”: on the left of each window is shown a linear combination of the fMRI maps that are well-correlated with finger tapping tasks (composite ICs 1 and 3), weighted by the *ΔHbR* part of the components at a specific point in time.** On the right of each window is shown the estimated *ΔHbR* components that are correlated with right finger tapping tasks. The time courses for IC1 (in blue) and IC3 (in green) are also plotted on the right of each window. Such a display provides a dynamic way to visualize the brain activity at different time points: −13460 ms **(A)**, −10452 ms **(B)**, −1082 ms **(C)**, 1747 ms **(D)**, 4045 ms **(E)**, 9527 ms **(F)** and 13768 ms **(G)**.

**Figure 7:**
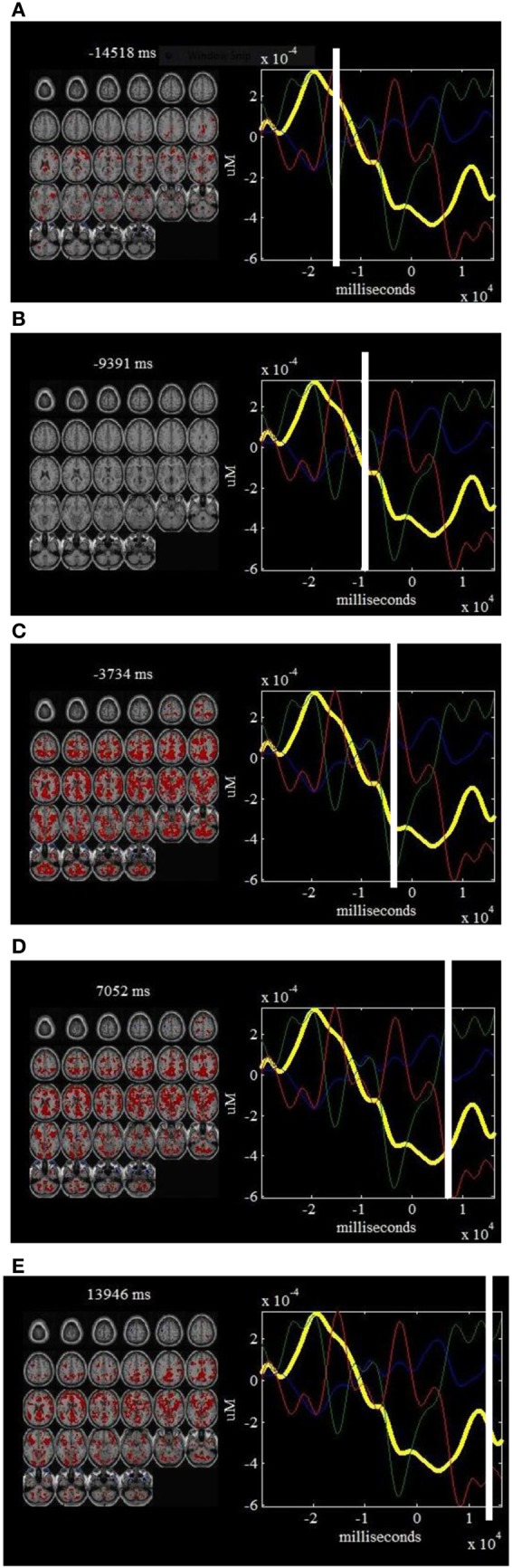
***ΔHbR* components and fMRI “snapshots”: on the left of each window is shown a linear combination of the fMRI maps that are well-correlated with physiology and body movement noise (composite ICs 2, 4, and 5), weighted by the *ΔHbR* part of the components at a specific point in time.** On the right of each window is shown the estimated *ΔHbR* components that are correlated physiology and body movement noise. The time courses for IC2 (in blue), IC4 (in green) and IC5 (in red) are also plotted on the right of each window. Such a display provides a dynamic way to visualize how the noise affects the brain activity at different time points: −14518 ms **(A)**, −9391 ms **(B)**, −3734 ms **(C)**, 7052 ms **(D)** and 13946 ms **(E)**.

Figure 4 shows seven “snapshots” snipped from Movie 1 produced from fused *ΔHbO_*2*_* and fMRI data for finger tapping tasks. On the left of the “snapshots” a linear sum of the fMRI maps weighted by their respective *ΔHbO_*2*_* time courses are provided while the mean *ΔHbO_*2*_* waveforms combined the estimated *ΔHbO_*2*_* components are plotted on the right of each “snapshot.” Figure 5 plots five “snapshots” captured from fused the fMRI and *ΔHbO_*2*_* data, which basically reveals the spatiotemporal responses of the physiology and body movement noise. Likewise, the “snapshots” from Movie 3, 4 produced from fused fMRI and *ΔHbR* components are given in Figures 6, 7 for finger tapping tasks and physiology and body movement noise, respectively.

## Discussion

We show, for the first time, a spatiotemporal reconstruction of the human brain's responses with high temporal and spatial resolution by fusing together the fNIRS signals and fMRI images. The jICA is a very key and unique technique for connecting hemodynamic fNIRS temporal signal and hemodynamic fMRI spatial information according to the joint constraints of temporal and spatial independences. Such direct correlation further provides a very valuable tool to localize non-invasively those high resolution structures that underlines temporally well-resolved fNIRS responses. Meanwhile, deep brain structures, which are not readily detectable with scalp fNIRS alone, can now be identified by the fusion technique when aided by the correlated spatial components from fMRI.

It is observed from Figure 2 that the first and third *ΔHbO_*2*_-fMRI* joint components basically identifies body movement and negative eye blink activities in pre-frontal lobe while the second, fourth and fifth *ΔHbO_*2*_-fMRI* joint components are fused with hemodynamic activity in left primary motor cortex (PMC), *supplementary motor area (SMA)*, and motor association cortex such as parietal cortex. In particular, the fifth joint component basically identifies the neural activity in the SMA and parietal cortex. These observations are further validated by Movies 1, 2, which show the dynamic interplay between space and time of *ΔHbO_*2*_*-*fMRI* hemodynamic responses. It is noted Movie 1 displays the spatiotemporal changes of neural activation patterns for finger tapping tasks. Further, we can see from Figure 3 that the second, fourth, and fifth *ΔHbR*-*fMRI* joint components basically reveal body movement and other physiology noise while the first and third *ΔHbR*-*fMRI* joint components identify the patterns of brain activity in the left motor cortex and motor association cortex. In particular, the first *ΔHbR*-*fMRI* joint component reveals brain activity in SMA and parietal cortex. These spatiotemporal findings from fused *ΔHbR*-*fMRI* data are further processed to generate Movies 3, 4, in which we can see the hemodynamic changes for a finger tapping task with high temporal and spatial resolution, and we can also see clearly how physiology and body movement noise corresponds to the neural activity. Importantly, we can observe from Figures 2, 3 as well as Movies 1, 3 that strong brain activity mainly occurs in the left PMC, which validates stimuli in the right finger tapping task will yield the activations in the left PMC.

Alternatively, we can see from Figure 4 the estimated *ΔHbO_*2*_* time courses (i.e., linear combinations of the significant *ΔHbO_*2*_* components) at specific positions in the cerebral cortex. The first “snapshot” in Figure 4A is associated with physiology and body movement noise while the second to fourth “snapshots” show the *ΔHbO_*2*_* concentration change during onset and stimulus processing of right finger tapping, which are validated by the spatial maps of fMRI image on the left. We can further observe from Figure 4 that the fifth “snapshot” captures the maximized spatiotemporal hemodynamic responses while the sixth and seventh ones show the decreased trends of neural activity. So if we examine the different “snapshots” in Figure 4, the spatiotemporal dynamics of the right finger tapping are revealed. In addition, the negative and positive peaks of the estimated *ΔHbO*_*2*_ time courses in Figure 5 directly correspond to the physiology and body movement noise though partly they capture the weak and negative hemodynamic responses of right finger tapping in the case of strong body movement.

The *ΔHbR-fMRI* “snapshots” in Figure 6 identify SMA and parietal cortex with early peak hemodynamic responses. Later responses occurring in left PMC show peaks of the *ΔHbR* responses during stimulus processing. In particular, “snapshots” 2–7 display the hemodynamic changes in detail with high spatiotemporal resolution for finger tapping task. Figure 7 provides the portions of estimated *ΔHbR* time courses at specific positions of fMRI images, in which we observe the peaks basically corresponding to the activities of physiology noise, eye blinks, and body movement.

In summary, we have used a combination of hemodynamic fNIRS and fMRI data to visualize the neural activation patterns with high spatiotemporal resolution. The fusion techniques have shown the potential by using the joint hemodynamic data to identify unique neural information that cannot be revealed in either technique alone. In particular, we implemented a joint decomposition of fNIRS and fMRI data, which was linked or fused by a common mixing parameter. The present method does not involve the solution of inverse problem for diffuse optical imaging or involve the use of the threshold for the fMRI data. However, we do need to decompose the combined data into specific components by using jICA, which are composed of fNIRS and fMRI portions. Separating the data into joint components will provide us a useful way to examine component specific differences among different patient groups or different stimulus tasks.

We have to point out the present investigation has significant limitations though we are encouraged that the results we have found seem meaningful and valid. For example, we only validate data from a single subject even without accurate measurements from fMRI. In future we would like to examine our method to incorporate different subject groups and complex neural stimuli as well as multiple time points acquired from multiple channels of fNIRS systems.

### Conflict of interest statement

The authors declare that the research was conducted in the absence of any commercial or financial relationships that could be construed as a potential conflict of interest.

## Supplementary material

Movie 1 ***ΔHbO2* and fMRI fusion movie for finger tapping tasks**.

Movie 2 ***ΔHbO2* and fMRI fusion movie for physiology and body movement noise**.

Movie 3 ***ΔHbR* and fMRI fusion movie for finger tapping tasks**.

Movie 4 ***ΔHbR* and fMRI fusion movie for physiology and body movement noise**.

## References

[B1] Bell A. J. Sejnowski T. J. (1995). An information-maximization approach to blind separation and blind deconvolution. Neural Comput. 7, 1129–1159 10.1162/neco.1995.7.6.1129 7584893

[B2] Brunno S. Luciano G. Konstantinos P. Pietro S. Massimiliano M. Simone C. (2011). An exploratory fNRIS study with immersive virtual reality: a new method for technical implementation. Front. Hum. Neurosci. 5:176 10.3389/fnhum.2011.00176 22207843 PMC3246589

[B3] Calhoun V. Adali T. Pearlson G. Kiehl K. (2006a). Neuronal chronometry of target detection: fusion of hemodynamic and event-related potential data. Neuroimage 30, 544–553 10.1016/j.neuroimage.2005.08.060 16246587

[B4] Calhoun V. Adali T. Liu J. (2006b). A feature-based approach to combine functional MRI, structural MRI and EEG brain imaging data, in Proceedings of the 28th IEEE EMBS Annual International Conference (New York, NY), 3672–3675 10.1109/IEMBS.2006.259810 17946195

[B5] Calhoun V. D. Adali T. Pearlson G. D. Pekar J. J. (2001). A Method for making group inferences from functional MRI data using independent component analysis. Hum. Brain Mapp. 14, 140–151 10.1002/hbm.1048 11559959 PMC6871952

[B6] Carpenter C. Pogue B. Jiang S. Dehghani H. Wang X. Paulsen K. D. (2007). Image-guided optical spectroscopy provides molecular-specific information *in vivo*: MRI-guided spectroscopy of breast cancer hemoglobin, water, and scatter size. Opt. Lett. 32, 933–935 10.1364/OL.32.000933 17375158

[B7] Cope M. Delpy D. T. (1988). System for long-term measurement of cerebral blood and tissue oxygenation on newborn infants by near infra-red transillumination. Med. Biol. Eng. Comput. 26, 289–294 10.1007/BF02447083 2855531

[B8] Cui X. Bray S. Bryant D. Glover G. H. Reiss A. L. (2011). A quantitative comparison of NIRS and fMRI across multiple cognitive tasks. Neuroimage 54, 2808–2821 10.1016/j.neuroimage.2010.10.069 21047559 PMC3021967

[B9] Egetemeir J. Stenneken P. Koehler S. Fallgatter A. J. Hermann M. J. (2011). Exploring the neural basis of real-life joint action: measuring brain activation during joint table setting with functional near-infrared spectroscopy. Front. Hum. Neurosci. 5:95 10.3389/fnhum.2011.00095 21927603 PMC3168792

[B10] Ferradal S. L. Eggebrecht A. T. Hassanpour M. Snyder A. Z. Culver J. P. (2013). Atlas-based head modeling and spatial normalization for high-density diffuse optical tomography: *in vivo* validation against fMRI. Neuroimage. [Epub ahead of print]. 10.1016/j.neuroimage.2013.03.069 23578579 PMC4433751

[B11] Gagnon L. Yucel M. Dehaes M. Cooper R. Perdue K. Selb J. (2012). Quantification of the cortical contributions to the NIRS signal over the motor cortex using concurrent NIRS-fMRI measurements. Neuroimage 59, 3933–3940 10.1016/j.neuroimage.2011.10.054 22036999 PMC3279595

[B12] Hoshi Y. (2003). Functional near-infrared optical imaging: utility and limitations in human brain mapping. Psychophysiology 40, 511–520 10.1111/1469-8986.00053 14570159

[B13] Huppert T. Diamond S. Franceschini M. Boas D. (2009). Homer: a review of time-series analysis methods for near-infrared spectroscopy of the brain. Appl. Opt. 48, 280–298 10.1364/AO.48.00D280 19340120 PMC2761652

[B14] Jobsis F. F. (1977). Noninvasive, infrared monitoring of cerebral and myocardial oxygen sufficiency and circulatory parameters. Science 198, 1264–1267 10.1126/science.929199 929199

[B15] Singh A. K. Okamoto M. Dan H. Dan I Jurcak V. (2005). Spatial registration of multichannel multi-subject fNIRS data to MNI space without MRI. Neuroimage 27, 842–851 10.1016/j.neuroimage.2005.05.019 15979346

[B16] Sui J. Adali T. Pearlson G. Clark V. Calhoun V. (2009). A method for accurate group difference detection by constraining the mixing coefficients in an ICA framework. Hum. Brain Mapp. 30, 2953–2970 10.1002/hbm.20721 19172631 PMC2733923

[B17] Tak S. Yoon S. J. Jang J. Yoo K. Jeong Y. Ye J. C. (2011). Quantitative analysis of hemodynamic and metabolic change in subcortical vascular dementia using simultaneous near-infrared spectroscopy and fMRI measurements. Neuroimage 55, 176–184 10.1016/j.neuroimage.2010.11.046 21094685

[B18] Ye J. C. Tak S. Jang K. E. Jung J. (2009). NIRS-SPM: statistical parametric mapping for near-infrared spectroscopy. Neuroimage 44, 428–447 10.1016/j.neuroimage.2008.08.036 18848897

[B19] Yuan Z. (in press). Spatiotemporal and time-frequency analysis of fNIRS brain signals using independent component analysis. J. Biomed. Opt. 10.1117/1.JBO.18.10.106011 24150092

[B20] Yuan Z. Zhang Q. Sobel E. Jiang H. (2010a). Image-guided optical spectroscopy in diagnosis of osteoarthritis: a clinical study. Biomed. Opt. Express 1, 74–86 10.1364/BOE.1.000074 21258447 PMC3005153

[B21] Yuan Z. Zhang Q. Sobel E. Jiang H. (2010b). High resolution x-ray guided 3D diffuse optical tomography of joint tissues in hand osteoarthritis: morphological and functional assessments. Med. Phys. 37, 4343–4354 10.1118/1.3467755 20879594 PMC2927690

